# A Person‐Centred Prehabilitation Program based on Cognitive Behavioural Physical Therapy for patients scheduled for Lumbar Fusion surgery: A mediation analysis to assess fear of movement (kinesiophobia), self‐efficacy and catastrophizing as mediators of health outcomes

**DOI:** 10.1002/ejp.2004

**Published:** 2022-07-18

**Authors:** Gemma Mansell, Marlies den Hollander, Hanna Lotzke, Rob J. E. M. Smeets, Mari Lundberg

**Affiliations:** ^1^ School of Psychology Aston University, Aston Triangle Birmingham UK; ^2^ Adelante Centre of Expertise in Rehabilitation and Audiology Hoensbroek The Netherlands; ^3^ Research School CAPHRI, Department of Rehabilitation Medicine Maastricht University Maastricht The Netherlands; ^4^ Back in Motion Research Group, Department of Rehabilitation, Ängelholm Hospital Ängelholm Sweden; ^5^ CIR Revalidatie Eindhoven The Netherlands; ^6^ Pain in Motion International Research Group (PiM); ^7^ Back in Motion Research Group, Department of Health Promoting Science Sophiahemmet University Stockholm Sweden; ^8^ University of Gothenburg Centre for Person‐Centred Care (GPCC), Sahlgrenska Academy, University of Gothenburg Gothenburg Sweden

## Abstract

**Objective:**

To investigate whether early changes in fear of movement (kinesiophobia), self‐efficacy and catastrophizing were mediators of the relationship between allocation to the pre‐habilitation intervention and later changes in health outcomes.

**Methods:**

The original pre‐habilitation trial (PREPARE, ISRCTN17115599) recruited 118 participants awaiting lumbar fusion surgery, half of whom received a prehabilitation intervention designed based on the modified fear‐avoidance model and half of whom received usual care. Mediation analysis was performed to test each mediator separately. Analysis was performed on each outcome of interest separately (Oswestry disability index, patient‐specific function, EQ general health and moderate/vigorous physical activity). Mediation analysis was carried out using PROCESS. Beta coefficients and bootstrapped 95% CIs were used to interpret the results.

**Results:**

None of the potential mediators was found to mediate the relationship between allocation to the intervention and 3‐month scores on any of the health outcomes tested.

**Conclusions:**

Screening patients for higher levels of catastrophizing and fear avoidance and lower levels of self‐efficacy could help ensure only the patients who are most likely to benefit from the intervention are included.

**Significance:**

Prehabilitation interventions for spinal fusion surgery have been found to improve health outcomes for patients. Theory‐based interventions that target key mechanisms are more effective at improving outcomes than non‐theory‐based interventions. While no mediating effects were found for this particular intervention, the analysis suggests that the underlying theoretical model and treatment targets are appropriate and could drive improvement if more strongly targeted.

## 1 INTRODUCTION

Over the past two decades, the number of lumbar fusion operations has increased worldwide, including in the USA and Sweden (Iderberg et al., 2019; Martin et al., 2019). The degenerative lumbar disease is the main reason for surgical intervention, however, outcomes after surgery for degenerative lumbar diseases are suboptimal, with persistent pain, poor function and poor quality of life in up to 40% of patients (Jonsson et al., 2017). Patients with chronic low back pain caused by degenerative disc disease (DDD) are relatively young (mean age 46 years according to the Swedish Spine Register), and thereby have a greater risk of more years lived with disability. Pre‐surgical psychological states, such as catastrophizing and fear of movement (kinesiophobia), are significant predictors of pain and poor function after lumbar surgery (e.g. Abbott et al., 2011; Mannion et al., 2007; Van Bogaert et al., 2022). Likewise, 70% of potential candidates for lumbar surgery report kinesiophobia (Kemani et al., 2020; Lundberg et al., 2009).

The time before surgery has been acknowledged to be a window of opportunity for behavioural change (Abbott et al., 2010; Santa Mina et al., 2015) and intervening prior to surgery (prehabilitation) to ensure the best possible outcomes has grown popular (Le Roy et al., 2016; Lundberg et al., 2019). Such interventions often include a psychological element or incorporate psychological techniques (Tong et al., 2020) and have been found to be effective in reducing anxiety during the post‐operative period (Tong et al., 2020). There is a lack of evidence for pre‐habilitation on spinal surgery outcomes specifically (Gometz et al., 2018).

Our pre‐habilitation RCT (Lotzke et al., 2016, 2019) addressed this gap by testing a pre‐habilitation intervention against conventional care for patients undergoing lumbar fusion surgery for degenerative disc disease. A modified version of the original fear‐avoidance model (Vlaeyen et al., 1995) was used as a framework for the intervention, which involved discussing and challenging patients' beliefs about physical activity, education about pain and activity, goal‐setting to increase activity and increasing confidence to resume physical activity post‐surgery. Lotzke et al. hypothesized that addressing self‐efficacy specifically as part of this intervention would help to reduce avoidance and increase activity and function. More detail about the intervention is given below and in Lotzke et al. (2016). The intervention was found to be effective in improving health‐related quality of life directly after the intervention but was no more effective than conventional care on outcomes such as disability 6‐month post‐surgery (Lotzke et al., 2019). However, between‐group changes in the psychological factors targeted in the intervention (catastrophizing, fear avoidance and self‐efficacy) occurred before this point, with changes seen at 3‐week, 8‐week and 3‐month post‐surgery.

A systematic review of seven mediation analysis studies found evidence for catastrophizing and self‐efficacy as mediators of pain and disability outcomes across a range of interventions for MSK pain (Mansell et al., 2013). Another systematic review of observational studies (*n* = 12) also identified self‐efficacy and fear avoidance as mediators of the relationship between pain and disability (Lee et al., 2015). A recent study (Fors et al., 2021) conducted a mediation analysis of a pre‐habilitation intervention in a group of patients with diagnosed degenerative lumbar spine disorder (*n* = 242), and found self‐efficacy as a mediator of the relationship between intervention allocation and disability, back pain intensity, and health‐related quality of life. Further testing of potential mediating variables specific to the model underlying our trial was warranted.

For the present analysis, based on the theoretical model and empirical findings, we expected changes in the potential mediators to occur early because they were being directly targeted as part of the intervention. We, therefore, hypothesized that early changes (3‐week post‐surgery) in kinesiophobia, pain catastrophizing and/or self‐efficacy would mediate the effects of the PREPARE pre‐habilitation programme on health outcomes at 3‐month post‐surgery.

## 2 MATERIALS AND METHODS

The AGReMA guideline (Lee et al., 2021) for reporting mediation studies was followed while writing this manuscript.

The PREPARE study (ISRCTN17115599) (Lotzke et al., 2016, 2019) was a prospective randomized controlled trial. Data were collected at baseline (between 12‐ and 8‐week pre‐surgery) and five further follow‐up points (1 week before surgery and 3‐week, 8‐week, 3‐month and 6‐month post‐surgery). In this study, the 3‐month post‐surgery assessment point was used. Participants were all awaiting lumbar fusion surgery, were adults (aged between 18 and 70 years) and had chronic LBP with additional minor radiating symptoms. The trial excluded those with previous surgery, spinal malignancy, dominating radiculopathy, neurological disorders or deformities in the spine or poor understanding of Swedish. They were recruited from three sites (one of two private spine centres or a university hospital in Gothenburg, Sweden) between April 2014 and June 2017. Eligible participants were randomized to either conventional care or an individual person‐centred ‘pre‐habilitation’ intervention which used cognitive behavioural techniques to target fear of movement and self‐efficacy in order to reduce disability post‐surgery. In the trial, ‘person‐centred care’ was defined as placing emphasis on recognizing the patient as a person with their own needs, will and feelings. The essence of person‐centred care is the partnership between the patient and the therapist, and together they formulate a health plan including goals and action plans to reach those goals (Ekman et al., 2011; Lotzke et al., 2016).

Mediation analysis is a statistical technique which can be used to identify whether a proportion of the treatment effect is transmitted through an indirect path. Indirect (or mediating) effects can help us learn more about how an intervention might achieve its effects, and indeed whether the intervention worked in the way it was hypothesized to. Mediation analysis can also be used when an intervention is found to be ineffective, to examine why this was the case (e.g. whether the hypothesized mediators did not change as expected) (O'Rourke & MacKinnon, 2018).

Mediation analysis of this trial was not specified during the development and analysis of the original trial, so no pre‐existing mediational hypotheses were made. However, a clear theoretical rationale for how the authors hypothesized the intervention might work was given, based on a modified version of the fear‐avoidance model (Lotzke et al., 2016). The authors further hypothesized that a patient's fear and catastrophizing also impacted their self‐efficacy for physical function (Lotzke et al., 2019). Self‐efficacy is a known important predictor of disability in patients with LBP (e.g. Alhowimel et al., 2018; Martinez‐Calderon et al., 2018).

Patients allocated to the PREPARE intervention received five 1‐h treatment sessions with a CBT‐trained physiotherapist, starting around 12–8 weeks before their surgery date and finishing with a ‘booster’ session 2‐week post‐surgery. The first session involved a cognitive interview to explore the patients' thoughts and feelings about staying active despite the pain. The second session was aimed at increasing patient knowledge about pain and activity and setting short‐term goals for increasing activity. The third session involved a cognitive behavioural experiment, where the patient was encouraged to test any negative expectations regarding physical activity. The fourth session aimed to increase self‐efficacy related to the short‐term goal set in session two and to set activity‐related goals to be reached post‐surgery. At each session, participants were given homework tasks to apply the techniques they have learned in the sessions.

Patients allocated to the conventional care arm were provided with basic information on the surgery they would receive, and the sort of exercises they would be expected to perform post‐surgery. They were encouraged to stay active according to standard procedures.

### 2.1 Measures

The outcomes of interest were functioning and health following surgery. The following measures were assessed as outcomes during the trial: the Oswestry Disability Index (ODI) (see Fairbank & Pynsent, 2000) (score between 0 and 100, higher score indicative of more severe disability); patient‐specific functioning (PSFS) (Stratford, 1995) (higher score indicative of higher functioning); general health (EQ VAS) (Connor‐Spady et al., 2015) and minutes of moderate to vigorous physical activity per day (MVPA), measured via an accelerometer. In the original trial, the ODI was used as the primary outcome, but in this secondary analysis, these additional functions and health outcomes were also analysed. The mediators of interest were exercise self‐efficacy, measured by the Exercise Self‐efficacy Scale (SEE) (Rydwik et al., 2014) (higher score indicative of higher self‐efficacy); fear of movement, measured by the Tampa Scale of Kinesiophobia (TSK) (Lundberg et al., 2004) (higher score indicative of higher fear avoidance) and pain catastrophizing, measured by the Pain Catastrophizing Scale (PCS) (Sullivan et al., 1995) (higher score indicative of more severe catastrophizing). The psychometric properties of these measures are described elsewhere (Lotzke et al., 2016), but in short, all measures included were found to be appropriate measures of each of the constructs of interest.

### 2.2 Effects of interest

**FIGURE 1:**
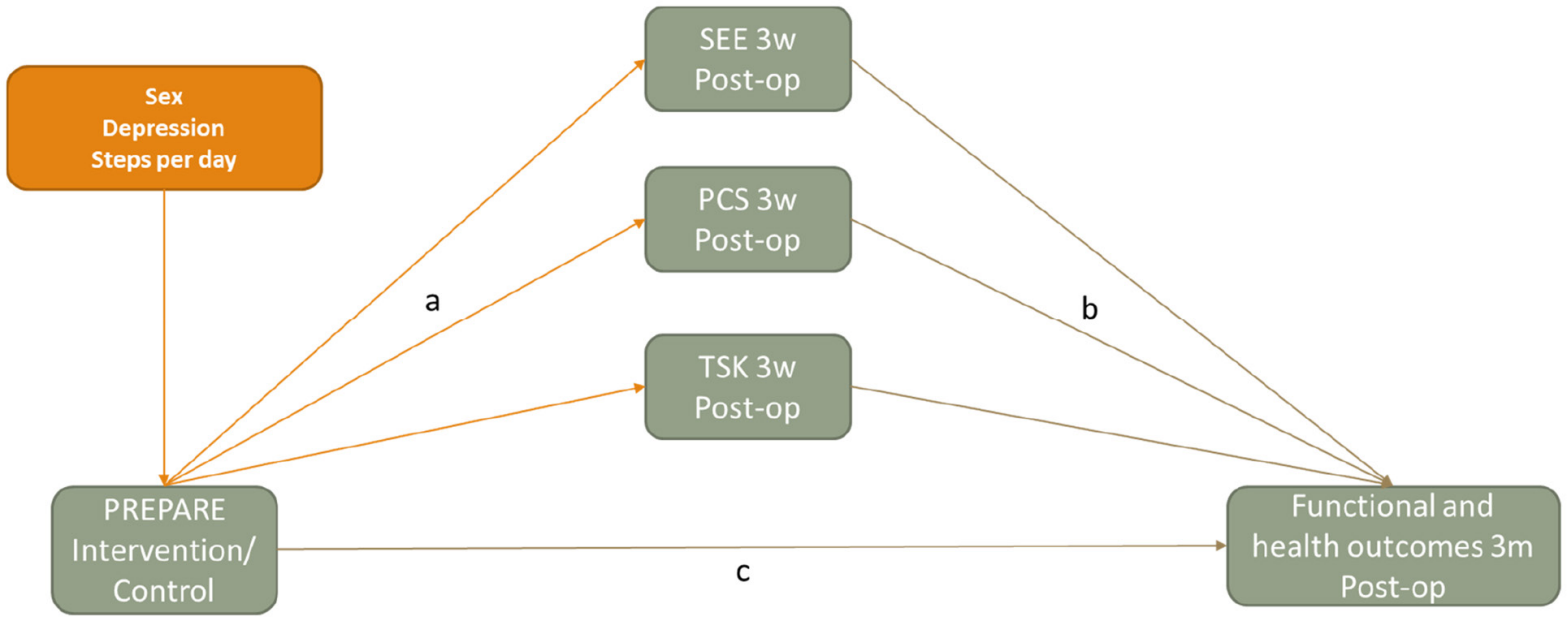
Directed acyclic graph (DAG) for the mediation analysis of the PREPARE intervention. Post‐op outcomes tested were disability (ODI); EQ5D Health VAS, patient‐specific functioning; moderate to vigorous physical activity. Mediation models were run separately for each mediator—shown in a single model here to save space.

We aimed to investigate whether fear of movement, self‐efficacy for exercise and pain catastrophizing mediated the effects of this pre‐habilitation program on physical activity and health outcomes at 3‐month post‐surgery. We estimated the unstandardized effect and corresponding estimates of uncertainty (95% CI) for both the *a* and *b* paths (intervention allocation to the potential mediator and potential mediator to outcome paths, respectively) (see Figure 1).

Potential confounding factors were identified as sex, depression score at baseline and steps per day at baseline. These were identified through discussions with clinical members of the research team as well as the use of a directed acyclic graph (DAG) (Tennant et al., 2020) to map these potential confounders (see Figure 1).

In the mediation model, the 3‐week post‐surgery assessment point was used for each of the mediators, and the 3‐month post‐surgery assessment point was used for the outcome. The 3‐week post‐surgery assessment point was chosen for the potential mediators because this was the point at which the whole intervention had been delivered, and participants had had time to apply some of the techniques following their surgery. The 3‐month post‐surgery assessment point was chosen for the outcomes because this was expected from a theoretical perspective, matched the primary assessment point in the original trial, and was the point at which change was seen in the original trial. Cases were not excluded on the basis of number of treatment sessions attended.

### 2.3 Statistical analysis

The framework employed to conduct this analysis was the product of the coefficients framework, from the ‘design‐based tradition’ (Preacher, 2015) of mediation analysis research. Mediation analysis was conducted using PROCESS (v3.5), accessed from https://www.processmacro.org/index.html. This software package allows for estimates of the direct, indirect and total effects and their bootstrapped 95% confidence intervals to be generated. PROCESS generates two linear regression models: the ‘mediator’ model with treatment allocation as the independent variable and each mediator as the dependent variable (*a* path) and an ‘outcome’ model with treatment allocation and each potential mediator as the independent variable and each outcome as the dependent variable (*b* path). All available data were used, including imputed data for the ODI, as per the original trial analysis (procedure can be found in Fairbank et al., 1980). While participants were recruited from three different sites, the majority of participants (90%) were recruited from one of the private clinics (Lotzke et al., 2019). No clustering was accounted for in the original analysis or in the present mediation analysis.

Mediation analysis requires us to make a number of assumptions regarding causality. Data from an RCT were used for this analysis, so it can be assumed that there was no confounding of the intervention–mediator and intervention–outcome effect paths. However, the mediator–outcome effect path is potentially confounded as both variables are outcomes of randomization. Potential confounding factors were therefore identified and included in the analysis. The assumption of no interaction between the intervention and mediator was accounted for by the PROCESS software, which includes an intervention–mediator interaction term. The temporal ordering of variables was based on the underlying theoretical model for the intervention (a modified version of the fear‐avoidance model), but the timing of the assessments limited the more nuanced ordering of the potential mediators (e.g. whether the change in fear of movement occurs before the change in self‐efficacy, as hypothesized by Lotzke et al., 2019).

Separate mediation models were constructed for each outcome and each potential mediator, and results were presented as both unadjusted and adjusted for the aforementioned confounding variables.

To further interpret the results of the mediation analysis, the action and conceptual theoretical framework were applied (Chen, 1990). In the context of an intervention study, ‘action’ theory (represented by the *a* path in the model) can give us an idea of whether the intervention was able to affect the proposed mediating variables, whereas ‘conceptual’ theory (the *b* path in the model) can give us an idea of how well the variables of interest fit the underlying intervention theory. Interpreting the estimates in each path can therefore help us understand more about the mediating effect itself (O'Rourke & MacKinnon, 2018).

Role of the funding source: The funders of the original trial played no role in the design, conduct or reporting of this study. The original trial was approved by the Regional Ethical Committee of Gothenburg, Registration number 586‐11, with amendment 527‐15.

## 3 RESULTS

In the original trial, 118 participants were recruited, with 59 randomized to the intervention arm and 59 to the usual care arm, and 54 participants from each group went on to receive surgery (Lotzke et al., 2019). By the 3‐month post‐surgery assessment point, 10 participants in the intervention group and 9 in the control group had been lost to follow up (17% and 15%, respectively). The mean age of participants was 46 years (SD 8.3), 53% were female, and a majority had completed at least high school education. Eighty‐seven (74%) of the participants had experienced LBP for >2 years, and 52 (44%) had experienced leg pain for >2 years. The mean scores for each outcome and potential mediator of interest are given in Table 1. Full details can be found elsewhere (Lotzke et al., 2019).

**TABLE 1:** Mean scores for each outcome and potential mediator of interest at baseline

Measure	Group	
	Active intervention	Conventional care
Disability (ODI)	35.7	38.0
Patient‐reported functioning (PSFS)	2.8	2.9
Pain catastrophizing (PCS)	22.5	23.1
Fear of movement (TSK)	37.8	38.5
Self‐efficacy for exercise (SEE)	61.5	60.8

### 3.1 Exercise self‐efficacy

Exercise self‐efficacy score at 3‐week post‐surgery was not found to be a mediator of any of the outcomes tested (3‐month ODI score, adjusted model −0.97, 95% CI −3.76 to 0.67; 3‐month PSFS score, adjusted model 0.10, 95% CI −0.24 to 0.42; 3‐month EQ General health score, adjusted model 0.53, 95% CI −1.38 to 3.37; three‐month MVPA score, adjusted model 1.05, 95% CI −0.77 to 3.59) (Table 2).

**TABLE 2:** Direct and indirect effects for each potential mediator and each outcome

Outcome: functional disability (ODI) at 3‐month post‐surgery	Exercise self‐efficacy (SEE) at 3‐week post‐surgery		Fear avoidance (TSK) at 3‐week post‐surgery		Pain catastrophizing (PCS) at 3‐week post‐surgery	
	Unadjusted B (95% CI) (*n* = 93)	Adjusted for sex, baseline depression score and baseline steps per day (*n* = 91)	Unadjusted B (95% CI) (*n* = 94)	Adjusted for sex, baseline depression score and baseline steps per day (*n* = 91)	Unadjusted B (95% CI) (*n* = 95)	Adjusted for sex, baseline depression score and baseline steps per day (*n* = 92)
Intervention mediator (path *a*)	8.16 (−1.03 to 17.34), *R* ^2^ 0.03	8.16 (−1.03 to 17.34), *R* ^2^ 0.03	−0.73 (−3.09 to 2.35), *R* ^2^ 0.00	−0.73 (−3.09 to 2.35), *R* ^2^ 0.00	−1.06 (−4.45 to 2.34), *R* ^2^ 0.00	−1.06, (−4.45 to 2.34), *R* ^2^ 0.00
Mediator outcome (path *b*)	−0.15 (−0.27 to −0.02), *R* ^2^ 0.05	−0.14 (−0.27 to 0.00), *R* ^2^ 0.04	0.72 (0.36 to 1.08), *R* ^2^ 0.14	0.78 (0.37 to 1.19), *R* ^2^ 0.14	0.63, (0.31 to 0.95), *R* ^2^ 0.14	0.60, (0.25 to 0.94), *R* ^2^ 0.12
Indirect effect (*ab*)	−1.31 (−4.19 to 0.46)	−0.97 (−3.76 to 0.67)	−0.62 (−2.76 to 1.89)	−0.08 (−2.08 to 2.86)	−0.80 (−2.70 to 1.80)	−0.43 (−2.15 to 2.49)

Outcome: patient‐specific function (PSFS) at 3‐month post‐surgery	Unadjusted B (95% CI) (*n* = 86)	Adjusted for sex, baseline depression score and baseline steps per day (*n* = 84)	Unadjusted B (95% CI) (*n* = 87)	Adjusted for sex, baseline depression score and baseline steps per day (*n* = 84)	Unadjusted B (95% CI) (*n* = 88)	Adjusted for sex, baseline depression score and baseline steps per day (*n* = 85)
Intervention mediator (path *a*)	8.16 (−1.03 to 17.34), *R* ^2^ 0.03	8.16 (−1.03 to 17.34), *R* ^2^ 0.03	−0.73 (−3.09 to 2.35), *R* ^2^ 0.00	−0.73 (−3.09 to 2.35), *R* ^2^ 0.00	−1.06 (−4.45 to 2.34), *R* ^2^ 0.00	−1.06 (−4.45 to 2.34), *R* ^2^ 0.00
Mediator outcome (path *b*)	0.01 (−0.02 to 0.03), *R* ^2^ 0.00	0.01 (−0.01 to 0.03), *R* ^2^ 0.00	−0.01 (−0.10 to 0.01), *R* ^2^ 0.03	0.00 (−0.07 to 0.08), *R* ^2^ 0.00	−1.70 (−0.07 to 0.07), *R* ^2^ 0.00	−0.06 (−0.12 to 0.00), *R* ^2^ 0.04
Indirect effect (*ab*)	0.08 (−0.31 to 1.42)	0.10 (−0.24 to 0.42)	0.52 (−0.45 to 1.48)	−0.01 (−0.22 to 0.13)	0.00 (−0.24 to 0.17)	0.07 (−0.16 to 0.33)

Outcome: EQ general health at 3‐month post‐surgery	Unadjusted B (95% CI) (*n* = 93)	Adjusted for sex, baseline depression score and baseline steps per day (*n* = 91)	Unadjusted B (95% CI) (*n* = 94)	Adjusted for sex, baseline depression score and baseline steps per day (*n* = 91)	Unadjusted B (95% CI) (*n* = 95)	Adjusted for sex, baseline depression score and baseline steps per day (*n* = 92)
Intervention mediator (path *a*)	8.16 (−1.03 to 17.34), *R* ^2^ 0.03	8.16 (−1.03 to 17.34), *R* ^2^ 0.03	−0.73 (−3.09 to 2.35), *R* ^2^ 0.00	−0.73 (−3.09 to 2.35), *R* ^2^ 0.00	−1.06 (−4.45 to 2.34), *R* ^2^ 0.00	−1.06 (−4.45 to 2.34), *R* ^2^ 0.00
Mediator outcome (path *b*)	0.12 (−0.00 to 0.28), *R* ^2^ 0.02	0.07 (−0.10 to 0.24), *R* ^2^ 0.10	−0.96 (−1.41 to −0.50), *R* ^2^ 0.16	−0.90 (−1.41 to −0.39), *R* ^2^ 0.12	−0.81 (−1.21 to −0.41), *R* ^2^ 0.15	−0.68 (−1.11 to −0.26), *R* ^2^ 0.10
Indirect effect (*ab*)	1.07 (−0.79 to 4.16)	0.53 (−1.38 to 3.37)	0.81 (−2.25 to 3.89)	0.09 (−2.86 to 2.78)	1.02 (−2.18 to 3.56)	0.49 (−2.97 to 2.44)

Outcome: MVPA at 3‐month post‐surgery	Unadjusted B (95% CI) (*n* = 84)	Adjusted for sex, baseline depression score and baseline steps per day (*n* = 82)	Unadjusted B (95% CI) (*n* = 85)	Adjusted for sex, baseline depression score and baseline steps per day (*n* = 83)	Unadjusted B (95% CI) (*n* = 85)	Adjusted for sex, baseline depression score and baseline steps per day (*n* = 83)
Intervention mediator (path *a*)	8.16 (−1.03 to 17.34), *R* ^2^ 0.03	8.16 (−1.03 to 17.34), *R* ^2^ 0.03	−0.73 (−3.09 to 2.35), *R* ^2^ 0.00	−0.73 (−3.09 to 2.35), *R* ^2^ 0.00	−1.06 (−4.45 to 2.34), *R* ^2^ 0.00	−1.06 (−4.45 to 2.34), *R* ^2^ 0.00
Mediator outcome (path *b*)	0.19, 95% CI −0.03 to 0.41, *R* ^2^ 0.03	0.12, 95% CI −0.05 to 0.28, *R* ^2^ 0.01	−0.10, 95% CI −0.76 to 0.57, *R* ^2^ 0.01	−0.13, 95% CI −0.66 to 0.40, *R* ^2^ 0.00	−0.12, 95% CI −0.74 to 0.50, *R* ^2^ 0.00	−0.17, 95% CI −0.30 to 0.65, *R* ^2^ 0.00
Indirect effect (*ab*)	2.03, 95% CI −0.16 to 5.68	1.05, 95% CI −0.77 to 3.59	0.06, 95% CI −1.18 to 1.63	−0.05, 95% CI −1.64 to 1.05	0.03, 95% CI −1.76 to 1.60	0.18, 95% CI −1.38 to 2.04

### 3.2 Kinesiophobia

Fear of movement score at 3‐week post‐surgery was not found to be a mediator of any of the outcomes tested (3‐month ODI score, adjusted model −0.08, 95% CI −2.08 to 2.86; 3‐month PSFS score, adjusted model −0.01, 95% CI −0.22 to 0.13; 3‐month EQ General health score, adjusted model 0.09, 95% CI −2.86 to 2.78 and 3‐month MVPA score, adjusted model −0.05, 95% CI −1.64 to 1.05) (Table 2).

### 3.3 Catastrophizing

Catastrophizing score at 3‐week post‐surgery was not found to be a mediator of any of the outcomes tested (3‐month ODI score, adjusted model −0.43, 95% CI −2.15 to 2.49; 3‐month PSFS score, adjusted model 0.07, 95% CI −0.16 to 0.33; 3‐month EQ General health score, adjusted model 0.49, 95% CI −2.97 to 2.44 and 3‐month MVPA score, adjusted model 0.18, 95% CI −1.38 to 2.04) (Table 2).

Estimates on the *a* paths (see Table 2) were generally larger but non‐significant (treatment allocation to self‐efficacy coefficient 8.16 [95% CI −1.03 to 17.34]; treatment allocation to fear‐avoidance coefficient −0.73 [95% CI −3.09 to 2.35] and treatment allocation to catastrophizing coefficient −1.06 [95% CI −4.45 to 2.34]). For the *b* paths (Table 2), estimates were negligible for PSFS (0.01 [95% CI −0.01 to 0.0.07] for self‐efficacy; 0.00 [95% CI −0.07 to 0.01] for fear avoidance and −0.06 [95% CI −0.12 to 0.00] for cactastrophizing), and larger but non‐significant for MVPA (0.12 [95% CI −0.05 to 0.28] for self‐efficacy; −0.13 [95% CI −0.66 to 0.40] for fear avoidance; −0.17 [95% CI −0.30 to 0.65] for catastrophizing). For the ODI (disability) and EQ (health) outcomes, the *b* path coefficients were more often statistically significant (ODI: −0.14 [95% CI −0.27 to 0.00] for self‐efficacy; 0.78 [95% CI 0.37 to 1.19] for fear avoidance and 0.60 [95% CI 0.25 to 0.94] for catastrophizing; EQ (Health): 0.96 [95% CI −0.10 to 0.24] for self‐efficacy; −0.90 [95% CI −1.41 to −0.39] for fear avoidance; −0.68 [95% CI −1.11 to −0.26 for catastrophizing]).

## 4 DISCUSSION

The present study used mediation analysis to test whether the effect of intervention allocation on health outcomes at 3‐month post‐surgery was mediated by fear of movement, exercise self‐efficacy or pain catastrophizing scores at 3‐week post‐surgery. We found none of these variables to be mediators of the effects of the pre‐habilitation intervention on health outcomes at 3‐month post‐surgery.

Action and conceptual theory provide a way of further interpreting the results of the mediation analysis (Chen, 1990; Lee et al., 2021). The non‐significant estimates on the *a* path appear to corroborate Lotzke et al's theory that the ‘dose’ of the intervention might not be strong enough for the intervention to be more effective than conventional care (Lotzke et al., 2019). The small, largely non‐significant estimates for the PSFS and MVPA outcome *b* paths suggest that the underlying theoretical model did not specifically change patient‐specific function or physical activity. The larger, statistically significant estimates on the *b* paths for the ODI and EQ outcomes suggest that targeting catastrophizing and fear avoidance was associated with improvement in these outcomes. Our results can therefore be interpreted as suggesting that the variables from the original fear‐avoidance model (Vlaeyen et al., 1995) are important to target, whereas self‐efficacy may be less important.

Our findings are inconsistent with Fors et al. (2021), who found that self‐efficacy was a mediator of ODI score. Like in Lotzke et al.'s study, the original trial by Lindbäck et al. (2018) also did not find a significant between‐group difference in ODI scores, but the intervention itself was based on several different theories and aspects of this may have been better able to target this particular factor. Fors et al. (2021) used to change between baseline and follow‐up rather than absolute scores for their analyses and tested multiple mediators in a single model rather than individually, which may also account for the difference in results. There is no one correct way to perform mediation analysis, and the method and technique chosen depend on the research question, study design and data available, but this could explain some of the differences in the results seen.

Our previous results were found to be non‐significant on the primary outcome variable (disability), but significant on secondary variables (health‐related quality of life). Understanding the level of treatment fidelity is crucial for interpreting an intervention's effectiveness. With non‐significant results and an unknown level of treatment fidelity, one cannot tell whether the problem was an ineffective treatment or a lack of treatment fidelity (Bellg et al., 2004). These mediation analyses suggest that the underlying theoretical model (conceptual theory—*b* path) targets appropriate variables, supporting the treatment fidelity of our active intervention.

As the comparator group in the original trial received conventional care, they would have had the opportunity to raise any concerns about activity post‐surgery with the physiotherapists and other healthcare professionals they consulted with. This could have been a key factor in helping to reduce fear of movement and catastrophizing, and increase self‐efficacy for exercise, which could have minimized the differences found between the two arms of the trial. Lotzke et al. (2019) acknowledged that physical therapists and surgeons in Sweden are aware of psychologically informed practice techniques, and one study found that Swedish surgeons recommend movement and activity post‐discharge more quickly than surgeons in the Netherlands (van Erp et al., 2018). Patients in the control arm of the Lotzke study could therefore still have received information to help reduce fear of movement and catastrophizing (albeit not as part of a structured intervention). Reassurance, and possibly therapeutic alliance, should be measured in future pre‐habilitation intervention studies to investigate the impact on outcomes.

The temporal ordering of the variables in this analysis is an important aspect of mediation analysis (Mansell et al., 2017). The 3‐week post‐surgery assessment point for the mediator was chosen because this was the point at which all treatment was completed, including the booster session. Within‐group change was seen in both arms by the first follow‐up point (1‐week pre‐surgery), particularly for fear of movement, but it was not possible to identify whether the change in fear of movement occurred before the change in self‐efficacy, as originally hypothesized in the trial, as improvements in both self‐efficacy and fear of movement scores were seen by the 1‐week pre‐surgery assessment point and continued to the 3‐week post‐surgery assessment point. Future trials should aim to assess key treatment targets at or after every treatment session and to identify which variables change when. This will provide evidence to improve theoretical models for future intervention development.

### 4.1 Limitations

The population included in the original trial included only a small/minor subgroup of patients who had high levels of fear of movement, high levels of catastrophizing and low levels of self‐efficacy, meaning that the intensive PREPARE intervention may not have been required for the majority of those who received it. This may have led to the minimal differences observed between groups in the original RCT and in the present mediation analysis. Another potential issue is the timing of the assessment points for the outcomes. The 3‐month post‐surgery assessment point was used for all outcomes to allow for comparison across the different measures, but it could be that for some outcomes, changes would not have occurred until after this point (e.g. Patient‐Specific Functioning Scale). Unmeasured confounding is a key issue in mediation analysis, even when randomization is present. Three key potential confounders were identified by the study team and controlled for in the analysis, but other unmeasured confounders, such as BMI, previous surgeries and medication use could also have had an impact on the results seen in the trial. Missing data were also an issue, with around 15% of data missing in each arm. Moreover, it is important to keep in mind that none of these results IS powered for mediation analyses, and a larger sample may have resulted in significant effects.

### 4.2 Implications

This study provides further insight into the PREPARE pre‐habilitation intervention by formally testing the hypotheses for how the underlying theoretical rationale using mediation analysis. While no mediating effects were identified, the results suggest that the underlying theoretical model is associated with functional improvement. Screening patients for higher levels of catastrophizing and fear avoidance, and lower levels of self‐efficacy, could help ensure only the patients who are most likely to benefit from the intervention are included.

## AUTHOR CONTRIBUTIONS

ML, RS, HL and MH had the original idea for the trial and collected the data used in this analysis. GM conducted the mediation analysis and drafted the manuscript. All authors discussed the results and commented on drafts of the manuscript. All authors approved the final draft for submission.

## FUNDING INFORMATION

No funding was secured for the present study. The original PREPARE RCT was funded with grants from AFA Research Funding No. 120216; Eurospine Research Grants No. TFR 8‐2014; the Swedish Research Council (VR) No. 2015‐02511; the Health and Medical Care Executive Board of the Västra Götaland Region (VGR) and Doctor Felix Neubergh grants.

## CONFLICT OF INTEREST

None declared.
